# Management of Uterine Fibroids and Its Complications During Pregnancy: A Review of Literature

**DOI:** 10.7759/cureus.31080

**Published:** 2022-11-04

**Authors:** Swarali G Datir, Arvind Bhake

**Affiliations:** 1 Pathology, Jawaharlal Nehru Medical College, Datta Meghe Institute of Medical Sciences, Wardha, IND

**Keywords:** benign tumors, fibroids, treatment, investigation, pregnancy

## Abstract

The word "uterine fibroids" itself indicates these are leiomyomas (a group of smooth muscle tumors that are benign), generally growing in the wall of the uterus. Nowadays, this is one of the most common diseases in women in the premenopausal age group. According to the studies, uterine fibroids are more commonly observed in women of African ancestry than white women. This article aims to educate readers on some fundamental concepts regarding uterine fibroids, such as the different types of fibroids according to various classification schemes, as well as their etiology, epidemiology, pathogenesis, management, and treatment, all of which must be understood by anyone studying medicine or working in the medical field. Along with reviewing current management options for uterine fibroids, it also identifies areas needing additional study to identify novel therapeutic targets and improve treatment individualization.

## Introduction and background

**Figure 1:**
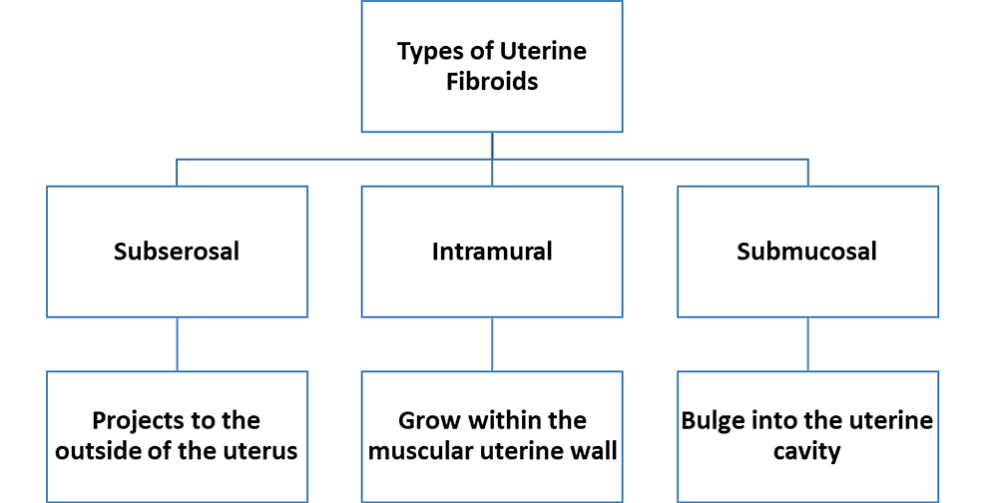
Types of uterine fibroids Figure credits: Swarali Datir (author)

The most prevalent type of benign tumor in smooth muscle cells of the uterus or the female reproductive organ is uterine fibroids. They are one of the most significant health problems worldwide for women, adversely affecting their physical and socioeconomic well-being [1]. Uterine fibroids severely impact one's health and quality of life [2]. Additionally, uterine fibroids tend to occur less frequently before puberty, more regularly during the reproductive years, and shrink in size after menopause in a woman's life cycle [3]. Uterine fibroids are classified into major three types according to their position (Figure 1), and their positions play a role in the present symptoms [4].

Menorrhagia, pelvic pain with or without dysmenorrhea or pressure symptoms, infertility, and recurrent pregnancy loss are the common symptoms that women with fibroids may experience. However, many times, it can be asymptomatic too. Most information that outlines the connection between the presence of fibroids and current symptoms is not confirmed yet. Even medical professionals and researchers have determined that the currently mentioned symptoms are the effects of myomectomy. Although there is no clear correlation between fibroids and infertility, an observational study (142 women) conducted in the USA found that the prevalence of fibroids in infertile women can be slightly high (i.e., 13%) [5]. Studies have shown that three characteristics can recognize uterine fibroids: increased smooth muscle cell proliferation, transformed extracellular matrix (ECM) deposition, and increased sensitivity to sex steroid hormones. The excess production and disorganized fibrous nature of ECM are the main characteristics of uterine fibroids [4]. Uterine fibroids are frequently discovered accidentally during routine bimanual pelvic or ultrasound examinations since they are typically asymptomatic or, to put it another way, rarely accompany symptoms [6]. A medical study conducted by Kirschen et al. [7] has shown a constant relationship between fibroids and systemic disorders, particularly those linked to endothelial or vascular dysfunction. Numerous exciting and vital questions concerning the causatives of uterine fibroids, whether fibroids are a uterine expression of a systemic illness, are raised by this recently discovered connection. Evidence suggests that fibroids are fundamentally related to vascular biology and may play a role in vascular dysfunction [7].

Studies show that after receiving therapy, the occurrence of new fibroids and the expansion of existing ones are sporadic [8]. Through altered gene expression, it has been possible to monitor the impact of fibroids on the endometrium. The immune system's environment and the body's vasoconstrictive components have also changed [9]. Since it is rarely observed, intra-abdominal bleeding, once thought to be a subsequent symptom of uterine fibroids, has become infrequent [10]. Research shows that women with fibroids experience cardiovascular risk factors more frequently than those without fibroids, especially body mass index (BMI) and hypertension [11].

## Review

Etiology and epidemiology

Leiomyoma growth is correlated with risk factors such as age, ancestry, family history, reproductive issues, sexual characteristics, hormones, hypertension, and contagion, consulting to epidemiology. Epidemiological research suggests that certain dietary elements and diet may have the power to impact hormone-related illnesses and perhaps even fibroid formation and development [1]. The most common tumor in women is uterine fibroids, which is generally observed in patients facing infertility complications [12]. The endogenous production of estradiol in fibroid tissue is permitted by aromatase, and fibroid stem cells have estrogen and progesterone receptors, which promote tumor development when these hormones exist [3]. The role of estrogen in this particular disease is essential [1].

In light of this, dietary practices and food ingredients may influence the chances of developing uterine fibroids. Indeed, contaminants found in foods such as fruits, vegetables, and fish may encourage several disorders linked to hormones. There is conflicting information regarding the connection between nutritional habits and the danger of uterine fibroids. Further research has revealed that low intakes of fruits and vegetables are connected to an advanced risk of fibroid formation and its development, vitamin D insufficiency is associated with an advanced risk of developing uterine fibroids, and contaminants consumed with food are connected to an advanced risk of developing uterine fibroids [1]. The unusual occurrence of intra-abdominal bleeding secondary to uterine fibroids continues to be poorly documented by clinicians [10]. Uterine fibroids may act as a barrier against endometrial cancer in females with gynecological diseases [13].

Risk Factor

Thyroid nodules were more commonly observed in patients with uterine fibroids than in people without them, indicating a possible positive association between them [14]. It is observed that risk factors for fibroids and cardiovascular disease overlap [11]. The most significant and commonly mentioned risk factors for uterine fibroids are age, the ancestry of Africans and Americans, early menarche, parity, and race. Recent risk factors include smoking, hormonal contraceptives use, alcohol consumption, and caffeine intake, having a high BMI, being premenopausal, having a family history of uterine fibroids, having hypertension, using dietary additives, and frequently drinking soya milk [15].

Pathogenesis

These tumors are produced by the myometrial tissue's proliferation and transformation under specific physiopathological circumstances [16]. There is a suggestion that uterine fibroid's pathogenesis resembles an injury response with the production of the rigid extracellular matrix; ischemia injury may be related to increased vasoconstrictive compounds during menstruation. However, in leiomyomas, vascular injury resulted in the overexpression of fundamental fibroblast development factors [1]. According to clinical research, the second phase of the cycle was when the proliferation markers in us were most highly expressed in tissue. Compared to typical uterine muscle cells, we have more sex steroid receptors. Progesterone's primary mode of action relies on the overexpression of cytokine-related genes and the elevation of specific development factor concentrations (such as transmuting growth factor β - TGF-β) within the tumor, which is similar to a self-stimulating method [17].

Management

Investigations

Pelvic examination: A tissue mass or enlarged uterus may be observed during a pelvic examination. An iron deficiency anemia diagnosis can be made if a woman reports heavy menstrual flow and suspected fibroids [18]. Ultrasound is the initial imaging mode of choice for fibroids [3]. Even with ultrasound, preoperative diagnosis is difficult in these cases [19]. Uterine fibroids can be found with transvaginal ultrasonography, which has a 90% to 99% sensitivity range; however, it can miss subserosal or tiny fibroids. Hysteroscopy or sonohysterography can increase the sensitivity of a test for submucosal fibroids. But even without pathologic analysis, there is no reliable way to distinguish benign from malignant tumors [3]. Imaging does not differentiate between fibroids and leiomyosarcoma nor reveals driver mutations or symptom presentation [2]. Saline infusion sonography, hysteroscopy, and magnetic resonance imaging (MRI) are the chosen imaging techniques for the analysis and description of uterine fibroids [20].

Treatment

The four significant aims in treating uterine fibroids are improving symptoms, decreasing fibroid size and maintaining the reduced size, conserving fertility if desired, and preventing damage. Uterine fibroids are prevalent, and only women with symptoms need treatment [21]. According to the research that has been published, vitamin D inhibits uterine fibroids growth, reduces its size, and improves symptoms [22]. Although hysterectomy has traditionally been the most prevalent therapy, modern management techniques offer various choices, from medical and surgical to radiological treatments. Each patient needs to receive treatment that is specifically required according to their symptoms, age, the number and position of their fibroids, and their desire to become pregnant in the future [23].

Although uterine fibroids are a universal severe health issue, the morbidity they cause is underscored because the top treatment choice for uterine fibroids is hysterectomy. This major surgical procedure removes the possibility of having children and has numerous adverse effects on general health. There are alternatives to hysterectomy for the treatment of uterine fibroid. Myomectomy, thermoablative therapies, blood vessel embolization procedures, magnetic resonance-guided focused ultrasounds (MRgFUSs), and indicative medical treatments are some more options for fibroids management. The therapeutic selection of treatments for uterine fibroids is mainly driven by the necessity to protect feminine productiveness and fibroid's unique characteristics [1].

Although it is observed that majority of women with fibroids are asymptomatic, few of them will present with significant symptoms that may include uncharacteristic uterine bleeding, anemia, pelvic pain and pressure, back pain, frequent urination, constipation, or infertility. Furthermore, poor obstetric results have been accompanied by fibroids. Interventional radiology treatments and expectant, medical, and surgical care are currently available options for treating symptomatic fibroid [24]. The goal is to offer significantly more efficient, secure, and specific medicines to the patient's particular tumor type [17].

Medical Treatment

Most medical treatments for fibroids prevent pregnancy, making them inappropriate for patients trying to conceive. Many medicines are now being investigated and may be effective in the context of fibroids in infertility. The only exclusion may be the preoperative usage of gonadotropin-releasing hormone agonists in anemias to limit hemorrhage and support developing hemoglobin levels.

Surgical Treatment

Hysteroscopy, laparotomy, laparoscopy, and robot-assisted laparoscopy are among the surgical procedures commonly used to treat uterine fibroids. Hysteroscopic techniques are chosen for submucous fibroids because they have a track record of producing successful results with minimal morbidity. A laparotomy is required when a fibroid is exceedingly large or when there is a suspicion of cancer. Still, an open procedure results in higher chances of blood loss, additional postoperative discomfort, and an extensive retrieval period. When compared to laparotomy, old-style and robot-assisted laparoscopy both produce identical pregnancy results, but they also have a markedly lower morbidity rate. When performed by a skilled surgeon, robot-assisted myomectomy can avoid many of the spatial challenges that conventional laparoscopy presents. However, the expense and increased operating time are the main drawbacks. A more thorough cost-effective investigation is required to determine the function of robot-assisted myomectomy in the arena of reproduction.

Other Treatment Methods

Although there are procedures for treating symptomatic uterine fibroids, such as uterine artery embolization and magnetic resonance-focused ultrasound surgery, there is some evidence that suggests these procedures may have unfavorable effects on pregnancy. However, reports of uncomplicated pregnancies with either of these approaches exist. In individuals with fibroids who want to get pregnant in the future, additional research is required to assess the well-being of uterine artery embolization and magnetic resonance-focused ultrasound operation [20]. Uterine artery embolization should not often be offered as a treatment of choice for uterine fibroids to women, fertile or infertile, who are pursuing a future pregnancy [25].

Since radiofrequency ablation (RFA) can be performed in a slightly invasive manner, it has become a safe and viable therapy option. RFA can be administered by a laparoscopic, transvaginal, or transcervical approach into a uterine fibroid to cause coagulative necrosis and improve symptoms associated with fibroid growth. RFA for uterine fibroids considerably lowers fibroid volume (Table 1), significantly improves the quality of life for those who have them, and is linked to low reintervention rates [26].

**Table 1:** Trend followed by volume of uterine fibroid under RFA procedure RFA, radiofrequency ablation

Fibroid volume reduction percentage	Timespan
0%	0 months
50%	3 months
60%	6 months
70%	12 months
80%	>12 months

The majority of fibroids are treatable endoscopically via laparoscopy or hysteroscopy. The surgeon must have experience with laparoscopic suturing. The best course of action for symptomatic women with uterine fibroids who desire to plan a family in the future is still laparoscopic myomectomy [27]. The established method of treating tumors is thermal ablation. Real-time thermal mapping and precision targeting have been made possible by the fusion of recently developed imaging techniques [28]. For women with us who want to become pregnant and give birth following the operation, MRgFUSs appears to be an intriguing least invasive option [29].

Complications related to pregnancy

Uterine fibroids are less likely to develop when contraception is used, especially in patients between 30 and 40 years of age. After taking oral contraceptive pills, the risk of fibroids attributable to favorable family history can also be reduced. The likelihood of developing uterine fibroids is increased when gynecological conditions, pregnancy, and gynecological procedures coexist [30]. Submucosal fibroids have been associated with advanced risks of miscarriage and lower rates of clinical pregnancy when it comes to pregnancy [31]. Even after correcting for numerous confusing variables, women with uterine fibroids discovered in the course of initial pregnancy have a great chance of developing hypertensive disorder in pregnancy (HDP) according to the study conducted by Chen et al. [32]. Pregnant females with uterine fibroids would have their blood pressure continuously observed, and HDP prevention procedures are required due to the harmful effects of HDP on pregnancy [32]. Uterine fibroids during pregnancy increase the chance of cesarean section, breech presentation, and postpartum hemorrhage [33]. The presenting fetal portion above the fibroid on term ultrasound signals indicates the requirement for cesarean delivery. An expert obstetrician may remove fibroids after a cesarean delivery (cesarean myomectomy); however, this is not usually necessary [31].

Only a decrease in birth weight for women with numerous fibroids provides clinical evidence for the theory that fibroids stunt fetal growth and lead to lower birthweight and earlier gestational ages at birth [34]. Both uterine fibroids and their therapies have the potential to result in irreversible infertility. Hence, surgical and pharmaceutical treatments to maintain or restore fertility are being researched, but, unfortunately, the success rate is very low [35]. For women who experience frequent miscarriages, infertility, or heavy uterine bleeding, hysteroscopic myomectomy is a recognized surgical procedure [27].

## Conclusions

In conclusion, substantial advancement has been achieved in the past 10 years in our understanding of the interactions between steroid hormones, danger issues, stem cells, genetics, and epigenetics that affect the pathophysiology and progressive source of uterine fibroids. A more comprehensive mechanistic understanding of the origin and complexity of uterine fibroids will result in long-term, fertility-friendly, and efficient drugs for treating patients with this most common tumor. Our understanding of fibroid growth and progress has improved, which makes it more and more obvious that fibroids do not present as independent entities different from the working of the remaining human body. The current unmet needs in this population include methods to enhance diagnosis and associated diseases, enhance fertility-preserving treatments, and enhance patient access and participation. Even its epidemiology has not yet been fully understood. Future research into the prevalent female condition that has a significant impact on women's health state will undoubtedly find this area to be exciting.
